# Analysis of the Provision of Plant‐Based, Vegetarian and Omnivorous Meals in UK Care Homes for Older People

**DOI:** 10.1111/jhn.70345

**Published:** 2026-10-08

**Authors:** Saffron Whyton, Lisa Methven, Kathrin Cohen Kadosh, Victoria Tischler

**Affiliations:** ^1^ School of Psychology University of Surrey Guildford UK; ^2^ Department of Food and Nutritional Sciences University of Reading Reading UK

**Keywords:** care homes, plant‐based diet, plant‐based protein, processed meat

## Abstract

**Introduction:**

With concerns growing around the impact of western diets on human and planetary health, a global transition is required towards primarily plant‐based diets. Although this subject has been the focus of increased research, the potential for meat and dairy reductions in care home settings has not been explored. This study assessed the provision of omnivorous, vegetarian and plant‐based meals in UK care homes.

**Methods:**

In total, 52 menus representing 136 care homes for older people were analysed against a scoring system that reflects the degree to which a menu is plant‐forward. Menus were scored out of 80 based on the number of plant‐based, vegetarian, red meat‐free and processed‐meat free meals provided across 1 week. A higher score indicates a greater proportion of plant‐based and vegetarian meals.

**Results:**

Menus scored on average 28, with a range of 19–41. The low score was primarily driven by a lack of entirely plant‐based meals and a reliance on red and processed meat‐based meals. Only two menus in the data set excluded processed meats, with all menus containing red meat at least once per week. Processed meats most commonly used in menus included sausages, cured meats and bacon. The most common plant‐based proteins included baked beans, lentils and mycoprotein.

**Conclusion:**

Even though the National Health Service recommends reductions in consumption of red and processed meats with increased consumption of plant‐based proteins, this transition is yet to be implemented in care homes. This study suggests that there is potential to improve the health and sustainability of meals provided in care home by increasing the availability of plant‐based options, especially those rich in plant‐based proteins.

## Highlights

- The present study explores provision of plant‐based, vegetarian and omnivorous meals in UK care homes.
- Fifty‐two care home menus were assessed using a novel scoring tool to determine the degree to which menus were plant‐forward.
- Menus scored an average of 28 out of 80, suggesting plant‐based meal provision is limited.
- On average, 8% of meals provided were plant‐based, whereas 16% of meals contained processed meat.

## 1 Introduction

### 1.1 Background

Data from the Food and Agricultural Organisation demonstrate that demand for livestock‐based foodstuff has risen globally, evident from the 27% growth in livestock between 1990 and 2021 [1]. Shifting our diets to reduce dependence on livestock for food intake could be beneficial for planetary and human health [2]. This is suggested by a growing body of evidence showing a shift towards plant‐based diets (PBDs), such as the planetary health diet [2], the healthy PBD or the Mediterranean diet. In 2025, the Eat‐Lancet commission published an updated report on healthy, sustainable and just food systems, with a key focus on the planetary health diet. The planetary health diet is characterised by increased fruit, vegetable, legume and wholegrain intake, and minimal meat, fish, eggs and dairy [2]. By transitioning towards the planetary health diet, approximately 15 million deaths per year could be averted due to a reduction in non‐communicable diseases such as cardiovascular disease, non‐insulin dependent diabetes and hypertension [2]. Furthermore, a transition to a mostly PBD could help achieve a 15% reduction in agricultural greenhouse gas emissions (GHGe) by 2050 [2]. Adherence to the healthy PBD could lead to reduced risks of developing frailty [3], dementia [4] and disabilities that affect activities of daily living [5]. Further to this, concerns about the health implications of red and processed meat consumption have led to both meat categories being classed as carcinogens, with recommendations to reduce intake to 90 g/day and 70 g/day, respectively [6]. Guidance from Cancer Research UK suggests red meat consumption should be limited to no more than three portions per week, with as little processed meat eaten as possible [7].

Despite the need for a global transition towards adherence to PBDs, institutions such as care homes (CHs) are often neglected from consideration. In the UK, per‐capita GHGe from food fell by 32% between 1986 and 2017 [8]. Limited evidence exists regarding both the sustainability of CH menus and the provision of animal‐based foods. In Denmark, a research study modelled a 25% decrease in GHGe for CHs within one municipality. Red meat, cheese and seafood were reduced in all modelled scenarios, with plant‐based proteins (PBPs) increased to compensate for the reduction in animal‐based foodstuff [9]. Further to this research, to meet previously proposed targets regarding GHGes, CHs in Denmark have reduced the meat content of their meals by 11%, but minimal efforts were seen to increase PBPs [10]. A study in a 144‐bed Italian CH found the majority of menus offered across the span of a year exceeded 2.03 kg of CO2. Furthermore, only 22% of the analysed menus were adherent to the planetary health diet [11]. In Canada, analysis of 32 CH menus found a significant number of calories came from animal‐based proteins (31.6%) with insufficient fibre, fruit and vegetables offered [12]. Fibre is commonly low in the diets of CH residents [13], despite the potential benefits of increased intake [14]. Healthy and sustainable meals may be limited in CHs for multiple reasons, including minimal staff education on nutrition and sustainability, conflicting interests with respect to ensuring autonomy and choice for residents, and barriers such as cost, labour shortages and availability of resources [15]. Working to increase PB meal offerings whilst simultaneously reducing red and processed meat could benefit both the resident's health as well as the planetary impact of CH menus.

### 1.2 Research Aims

The overarching aim is to explore the degree to which menus in UK care homes (CHs) for older adults are plant‐forward. For the purpose of this study, plant‐based meals are defined as those that do not contain any animal foodstuff, including dairy, eggs, meat and fish. Vegetarian meals exclude meat and fish but include dairy and eggs. Additional objectives include:

1. To explore the provision of red and processed meats in CHs.
2. To explore the provision of plant‐based protein in CHs.
3. To perform an exploratory analysis into the relationship between the demographics of CHs and the degree to which a menu is plant‐forward.

## 2 Materials and Methods

### 2.1 Study Overview

The study replicates an analysis of hospital menus in the UK using a novel scoring system [16]. The analysis in the present study focused on meals served at lunch and dinner, with breakfast excluded due to heterogeneity in the method of provision. Desserts were also excluded as they tended to be vegetarian. Starters at lunch were included in analysis, although provision of starters did vary across the data set. Furthermore, menus specifically designed to cater for specialised diets such as renal, diabetic, gluten‐free or pureed diets were excluded. Menus that exclusively met dietary preferences, including PB menus, were also excluded, due to high heterogeneity of provision (only one CH disclosed a fully PB alternative menu).

### 2.2 Recruitment and Data Collection

CHs in each region were identified through databases provided by the Care Quality Commission, Care Inspectorate (Wales and Scotland) and Regulation and Quality Improvement Authority (Northern Ireland). UK CHs which specialise in care for older adults were contacted and asked to provide a copy of their 7‐day lunch and dinner menus. The sample aimed to be representative of the UK, with a proportional number of CHs contacted from each county. Overall, 1000 independent CHs and CH management companies were contacted. CHs were contacted either directly via email, or newsletters circulated by ENRICH (Enabling Research in Care Homes) networks. ENRICH are funded by the National Institute for Health Research (NIHR) with the aim to promote awareness and participation in research for people who live and work in CHs. Additional information sought from each CH included the menu rotation length and the method of preparation (in‐house chef or external provider). Information derived from publicly available resources included the CH funding structure (for/non‐profit); whether privately owned, run by a local council or charity; and geographical location. Data were provided by individual CHs, or by the parent company (if part of a care home group), who shared a menu that represented all CHs within their group. Once menus were received, one researcher extracted the required information from the menus.

### 2.3 Calculating Total Scores

The scoring system was modified from a previously developed system with the authors' permission [16]. The scoring system was used to rate the CHs based on the provision of meat, dairy and plant‐based meals. The total score calculated from the scoring system was based on the summary of weighted sub‐scores (percentage provision of a category) (see Table 1). Sub‐scores for PB meals and daily PB options were assigned a greater weighting. Each score is based on a scale 0–80, where the highest score attainable is when all meals are PB and contain PBP.

**Table 1:** Novel scoring system developed in collaboration with key experts from Plant‐Based Health Professionals.

Meal component	Description	Range (%)	Weighting	Argument for weighting/inclusion
% of plant‐based meals	Meals not containing dairy, fish, eggs, honey or meat	0–100	2	Plant‐based meals are most likely to be environmentally friendly and a richer source of fruit, vegetables, legumes and wholegrains
% of meals not containing processed meat	Meals not containing cured meats, salami, bacon, ham, sausages, corned beef, jerky, canned meats, pate	0–100	1	Both red and processed meats are classed as carcinogens. Excluding these meats from meals is therefore important for health as well as the environment
% of meals not containing red meat	Meals that do not contain beef, lamb, mutton, pork, veal, venison and goat	0–100	1	
% of pescatarian meals	Meals that don't include meat, but can include fish, dairy and eggs	0–100	1	Fish and other seafood have a lesser environmental footprint than meat and potential health benefits
% of vegetarian meals	Meals that do not include meat and fish.	0–100	1	
% of meals containing a plant‐based protein	Includes nuts, seeds, tofu, legumes, beans. This can include a meal that uses a mix of plant‐based and animal‐based protein	0–100	1	Plant‐based protein is important for health as it is not only rich in protein, but fibre too. Both nutrients are important for older adults to obtain
At least one plant‐based option at both dinner and lunch		0 = No 50 = one meal 100 = Yes	2	Provision of a daily plant‐based option provides greater opportunity for health and environmental benefits compared to meat and dairy‐based dishes
At least one vegetarian option at both dinner and lunch		0 = No 50 = one meal 100 = Yes	1	

To calculate the final score, the percentage of meals not containing red and processed meat was used. To provide greater clarity, a worked example is provided below:

Step 1: Calculate percentage provision for each scoring category (see Table 2)

**Table 2:** Raw subscores (% provision) for Care Home 1.

% PB	% Vegetarian	% Pescatarian	% Not red meat	% Not processed meat	% PBP	Daily PB meals	Daily vegetarian meals
13	44	25	81	94	4	0	50

Step 2: Apply weightings (see Table 3)

**Table 3:** Weighted subscores for Care Home 1.

% PB	% Vegetarian	% Pescatarian	% Not red meat	% Not processed meat	% PBP	Daily PB meals	Daily vegetarian meals
26	44	25	81	94	4	0	50

A weighting was applied to PB categories in order to reach a total of 10 (see Table 1). Where PB meals were offered, the number of PB options and daily PB offerings were doubled. Weightings are applied so the weightings add up to 10.

Step 3: Multiply according to scoring system formula and divide by 10

$$\mathrm{Total}\;\mathrm{score}=\frac{26+44+25+81+94+4+0+50}{10}.$$

### 2.4 Statistical Analysis

Statistical analysis was performed using RStudio. Descriptive statistics and distributions were calculated for selected sub‐scores including for % PB meals and % meals containing PBP. Relationship between characteristics of the CHs and menu scores was calculated using Mann–Whitney *U* and Kruskal–Wallis tests due to a non‐normal distribution.

### 2.5 Ethical Considerations

This study did not require ethical review due to the absence of human participants. The project was registered with the University of Surrey Ethics Committee (Review Reference: 2025‐2592/2494‐1).

## 3 Results

### 3.1 Characteristics of Care Homes

In total, 52 menus were collected and used in the analysis. This represented 136 CHs in the United Kingdom (see Table 4).

**Table 4:** Characteristics of Care Homes included.

Factor	Number of care homes
Geographical location	South East (60), South West (24), Scotland (14), West Midlands (8), Wales (7), East (7), North West (5), East Midlands (4), London (3), Yorkshire and The Humber (3), Northern Ireland (1)
For profit/non‐profit	For profit (124), non‐profit (12)
Independent/part of group	Part of a care group (118), independent (18)
Method of meal provision	In‐house chefs (133), external provider (2), unknown (1)
Number of beds	51+ (63), 30–50 (63), 1–29 (10)
Average length of menu rotation	4 weeks (121), 3 weeks (5), 2 weeks (4), 5 weeks (3), unknown (3)

*Note:* Geographical location split by government office regions.

### 3.2 Care Home Menu Scores

The overall distribution of scores is denoted in Figure 1. Per menu, the average total score was 28 (range: 19–41). When weighted based on the number of CHs represented per menu, the average score rose to 30. Figure 2 expresses the percentage of meals in each dietary category, as well as denoting the number of meals containing PBP which was used to calculate the final score. Only two of the 52 menus did not include processed meats. Eight menus did not include any PB meals, and one did not include vegetarian meals. Only 15 of the 52 menus featured PB meat alternatives such as mycoprotein or soy burgers. Within those CHs, provision of meat alternatives ranged from one to four servings per week.

**Figure 1:**
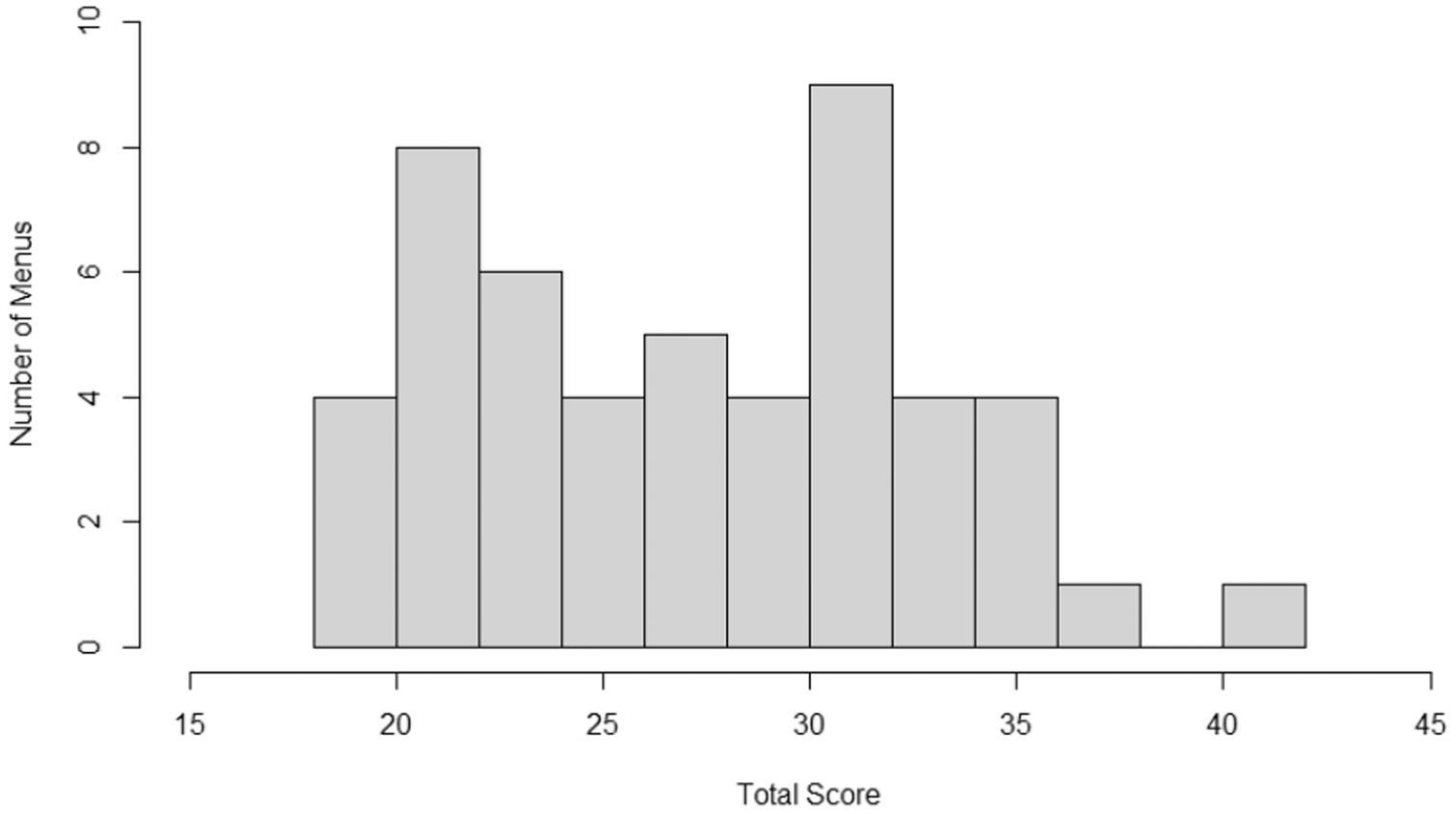
Distribution of total scores calculated using the scoring system across the data set.

**Figure 2:**
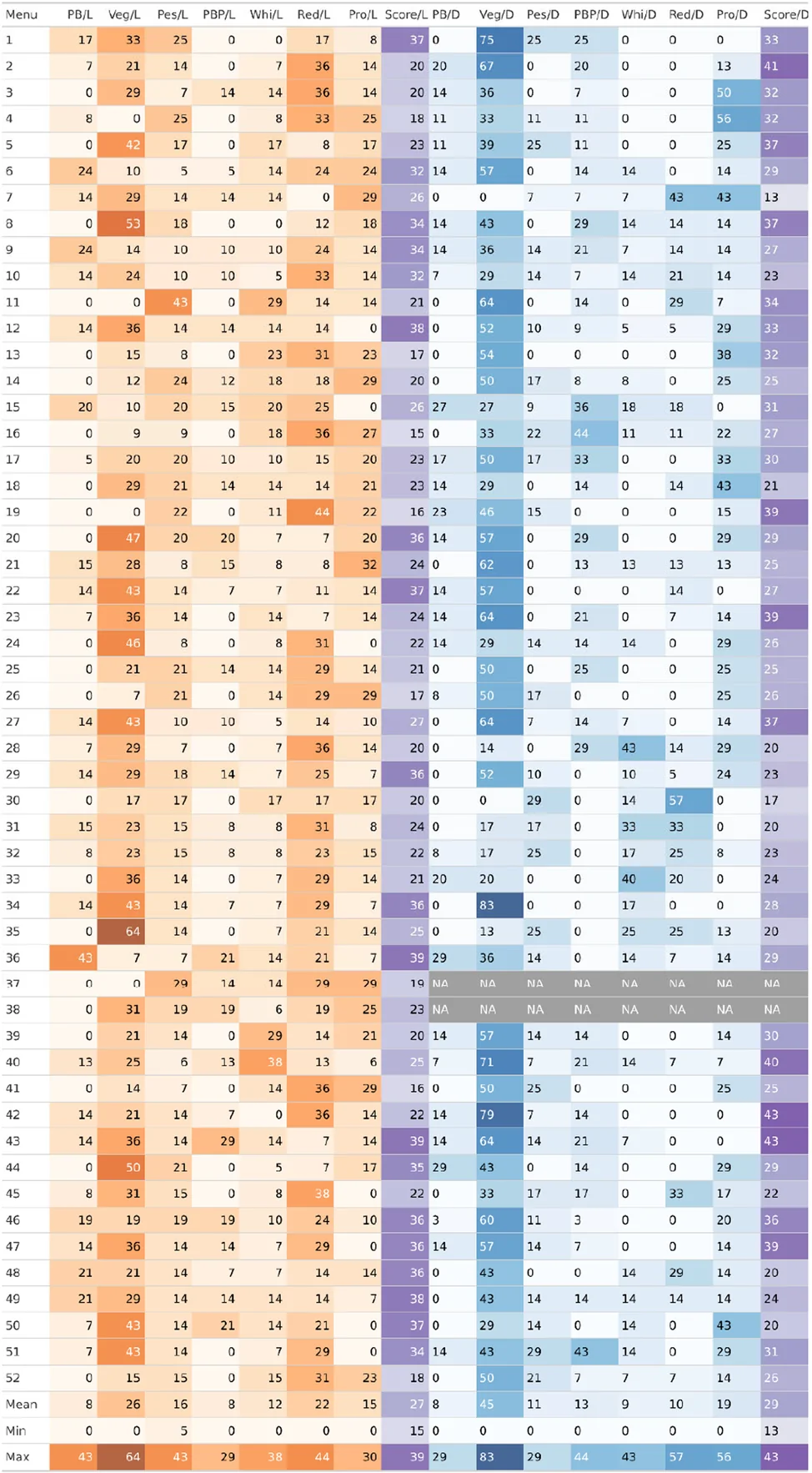
Total scores and % of meal provision for each CH menu per lunch and dinner: Lunch and dinner scores of unweighted menus. PB = plant‐based, PBP = plant‐based protein. Lunch is denoted in orange, dinner denoted in blue and total scores denoted in purple. Menus 37 and 38 did not provide dinner menus.

The provision of PB meals was limited across the data set (see Figure 3). On average, 8% of total meals were PB.

**Figure 3:**
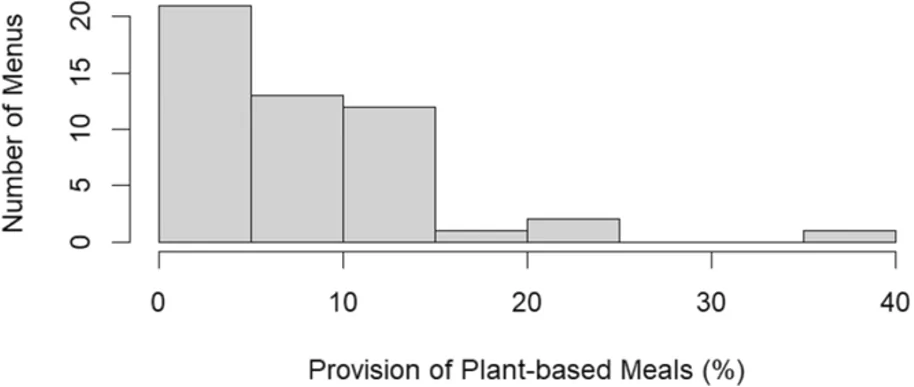
Provision of plant‐based meals per week.

Provision of PBP was limited across the data set with six menus not offering any source of PBP (Figure 4). On average, 7% of meals contained PBP. Pulses and mycoprotein were more likely to be the main protein offered, whereas baked beans were commonly used as a side dish alongside meat. Amongst the subgroups of PBP, baked beans were most commonly offered (57%), with mycoprotein (14%) and pulses (10%) also offered regularly.

**Figure 4:**
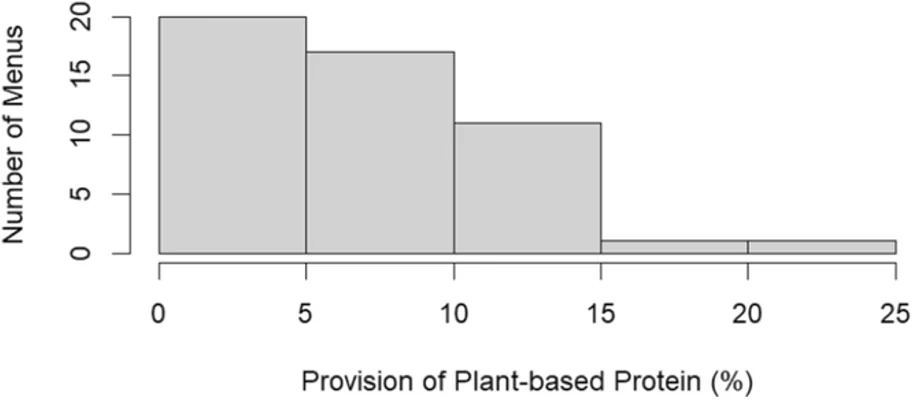
Provision of meals containing plant‐based protein per week.

In line with recommendations from Cancer Research UK [7], 37% of the menus had 3 portions or less of red meat offered throughout the week. However, 17% of meals on average contained red meat. The average menu consisted of 16% processed meat meals, with one menu using processed meats in 38% of their meals (Figure 5). Sausages were the most commonly provided processed meat, representing 40% of all offerings. As well as this, cured meats (such as gammon) (14%) and bacon (14%) were also offered frequently.

**Figure 5:**
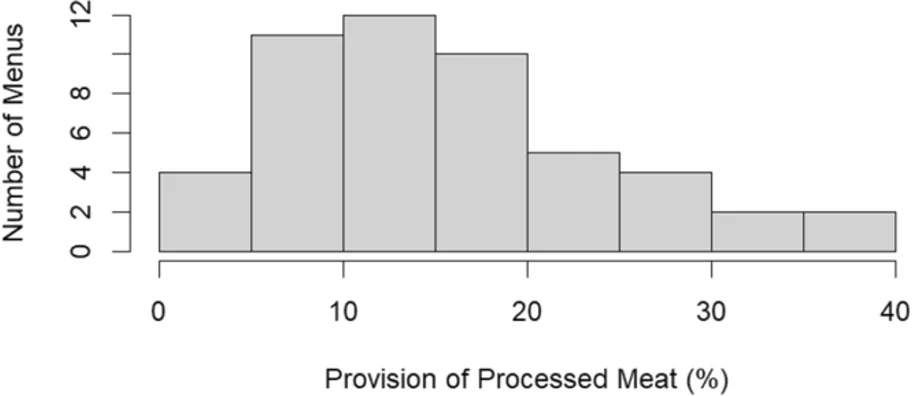
Provision of meals containing processed meat per week.

### 3.3 Relationship Between CH Type and Menu Scores

Location, size and management structure were compared to dietary meal provision and overall menu scores. Location was significantly associated with total score (*p* = 0.003) with West Midlands CHs scoring highest and Northern Irish CHs scoring lowest (32.7 vs. 19.3, respectively). Percentage PBP (*p* = 0.007), % red meat (*p* = < 0.001) and % vegetarian (*p* = < 0.001) were also associated with location, but % processed meat and % PB were not. For management structure (whether independently managed or owned by a parent company), only % PB meals (*p* = 0.01) and % processed meals (*p* = 0.001) were significantly associated. Independent companies had higher % of processed meat meals on their menus (19.4% ± 9.7% vs. 11.6% ± 6.0), whilst simultaneously offering a lower proportion of PB meals (8.1% ±9.8% vs. 10.7% ± 4.3%). CH size was associated with all measured factors (see Table 4). Provision of PB meals and processed meat were most strongly associated with CH size, with smaller CHs (1–29 beds) associated with higher processed meat provision and lower provision of PB meals. Only % PBP provision was associated with whether a CH was for or nonprofit (For 6.2% ± 3.9% vs. Non 8.3% ± 4.1%, *p* = 0.037) (see Table 5).

**Table 5:** Demographics of the data set compared to percentage provision and total scores.

	% Plant‐based protein	% Plant‐based	% Vegetarian	% Red meat	% Processed meat	Total score
Location						
East	6.1 (4.7)	12.0 (2.6)	44.0 (7.8)	11.0 (4.3)	13.7 (5.4)	31.7 (2.1)
East Midlands	7.2 (4.4)	7.5 (2.4)	38.5 (7.8)	19.3 (12.2)	14.7 (15.1)	28.8 (5.8)
London	6.4 (3.0)	9.4 (3.9)	33.3 (14.9)	13.4 (4.3)	23.5 (12.1)	28.9 (6.3)
Northern Ireland	7.1 (0)	4.8 (0)	9.5 (0)	28.6 (0)	38.1 (0)	19.3 (0)
North West	3.3 (3.0)	6.2 (6.0)	28.3 (15.9)	22.3 (11.1)	10.7 (7.8)	23.8 (5.5)
Scotland	8.8 (2.4)	13.7 (5.7)	25.9 (5.1)	25.2 (4.2)	13.9 (1.3)	28.7 (1.5)
South East	6.1 (3.8)	10.8 (5.4)	40.4 (7.8)	14.6 (4.3)	11.0 (5.5)	30.9 (4.4)
South West	7.0 (3.5)	9.7 (2.5)	40.6 (4.0)	14.5 (3.3)	12.0 (3.9)	31.7 (3.2)
Wales	3.4 (1.8)	9.6 (5.1)	41.9 (3.5)	11.5 (4.1)	13.0 (10.8)	32.5 (4.0)
West Midlands	3.9 (2.3)	9.3 (5.8)	42.6 (8.7)	15.1 (5.5)	11.7 (8.9)	32.7 (5.1)
Yorkshire	13.1 (7.8)	11.1 (8.2)	21.7 (18.4)	18.8 (8.2)	12.8 (12.3)	27.1 (6.4)
*p*	0.007	0.161	< 0.001	< 0.001	0.054	0.003
Management						
Group	6.4 (3.7)	10.8 (4.3)	38.4 (9.2)	15.9 (6.1)	11.6 (6.0)	30.7 (4.2)
Independent	5.9 (5.8)	8.2 (9.8)	32.3 (14.8)	16.7 (8.1)	19.4 (9.8)	28.1 (6.7)
*p*	0.343	0.011	0.117	0.406	0.002	0.119
Care home size						
Small	7.9 (6.4)	4.4 (4.1)	29.3 (14.1)	16.1 (6.4)	22.2 (9.1)	25.3 (4.9)
Medium	6.1 (4.1)	10.6 (5.5)	36.3 (9.9)	17.5 (6.9)	12.5 (6.6)	30.3 (4.9)
Large	6.4 (3.4)	11.2 (4.6)	40.0 (8.9)	14.9 (5.8)	11.1 (5.9)	31.2 (3.8)
*p*	0.05	< 0.001	0.0	0.019	< 0.001	0.005

*Note:* Value(value) represents mean(standard deviation).

## 4 Discussion

This study explored the provision of meat‐based, vegetarian and PB meals in CHs. Overall, all CH menus scored relatively low for weekly PB meals. Despite National Health Service (NHS) recommendations to reduce red and processed meats due to health and environmental concerns, over 99% of the CHs included in the data set used processed meat on their menu. Additionally, all menus contain at least one meal that features red meat. This is despite the fact that red and processed meats have been classed as possible and probable carcinogens, respectively [6].

### 4.1 Meat Provision in Care Home Settings

Currently, there is limited evidence exploring the provision of meat, fish and dairy in CHs. It has been suggested that the primary source of protein in CHs is dairy products [12, 17] with meat also contributing to a significant amount of total protein intake [17]. Research suggests that residents consume 1–3 portions of dairy foods per day [18]. PBPs contribute a significantly smaller amount to a resident's energy intake compared to animal‐based proteins [12]. Reflecting this lack of PBP, a study in an Italian CH suggested adherence to the planetary health diet was limited [11]. Furthermore, a systematic review found that fibre inadequacies were highly prevalent, with menus offering only 47% of recommended requirement [19]. In a study of 20 Slovenian CHs, high saturated fat intake was found to be associated with low fibre intake [20]. This could suggest a lack of plant‐forward meals as saturated fats are commonly found in meat and dairy, whilst fibre is derived from plants. There are many factors that influence the promotion and use of animal foods in CHs. These could include cultural relevance, familiarity, ease of preparation, cost and hedonic preferences [21], however, the motivations behind the use of animal foods in CHs are yet to be explored. A further reason to provide animal foods is the notion that they represent an important role in the health and nutrition of CH residents. Older adults are more vulnerable to undernutrition [22], and therefore interventions may be necessary in CHs to overcome nutritional inadequacies. Many common fortification methods involve the additional of full‐fat milk, milk powders and butter to foods [23]. Furthermore, milk, red meat and fish are noted as important sources of dietary minerals in CHs [24]. Recommendations to limit processed meats still extend to CH settings despite the potential protein contributions [23]. Additionally, the British Dietetics Association suggests swapping butter for PB alternatives, such as margarine and oils [23].

### 4.2 Feasibility of Moving Towards Plant‐Forward Menus

Outside of the CH context, PB meals offered on menus have outperformed omnivorous alternatives on nutrition and sustainability [25]. This paper suggests that PBMs provide both greater nutritional quality and lower environmental impact [25]. Despite the potential benefits, lack of staff and resident acceptance of PB meals could limit their uptake. Although many older adults are willing to consume PBPs, some opt to stick to what's familiar, citing a lack of motivation to consume PBPs [26]. Meal tasting sessions, staff education and chef training would be necessary to facilitate both the acceptance and uptake of PB meals [27]. Further research is needed to understand the feasibility of implementing PB meals onto CH menus.

### 4.3 Comparison of the Novel Scoring System to the Literature

This study is the first to adapt a novel scoring system for CH menus which assess the degree to which a menu is plant‐forward. Similar checklist‐style scoring systems have been employed in other settings. For example, one study used a seven‐domain checklist that assessed health and sustainability of school menus and compared this with policy requirements [28]. It was reported that none of the school canteens ensured three‐quarters of the plate was PB, and that there was a lack of legumes used as a primary protein source [28]. Another reported approach is using the Global Diet Quality Score metric, which was adapted to analyse the healthfulness of school menus [29]. The Global Diet Quality Score divides 25 food groups into healthy (i.e., deep orange vegetables), unhealthy if consumed excessively (i.e., red meat), and unhealthy (i.e., processed meat) and scores them based on the quantity and frequency of their offering. This system also positively scores for fortified foods. Similar to the novel scoring system employed in this study, the Global Diet Quality Score is counted numerically on a scale of 1–100. Another similar scoring system is the Childrens Menu Assessment used for menus targeting children. The Childrens Menu Assessment provides information on the availability of healthy options by scoring meal options based on their healthfulness. This tool is based on proportions of healthier foods such as salad‐based dishes and wholegrains, to unhealthy foods such as sugar‐sweetened beverages. The resulting score is between −5 to 21 [30]. Menu sustainability is often measured using specific metrics such as water and carbon footprints [31] or scoring indexes such as the Menu Sustainability index [32]. The healthfulness of a menu is often measured using nutrient profiling systems such as the Nutrient Rich Food Index [33] or other quantitative tools such as the nutriRECIPE index [34]. These tools analyse nutrients instead of foodstuffs. Other indexes, similar to the system used in the present study, seek to compare menu offerings to healthy diets, for example, the Mediterranean Diet Index assesses compliance with a Mediterranean diet whilst also considering portion size and menu variety [35].

### 4.4 Strengths and Limitations

The novel scoring system employed in this cross‐sectional study has several strengths and weaknesses. A key strength is the ease at which the scoring system can be employed. This meant the scoring system could be applied on a large scale across a variety of menus, with minimal input from the CHs. As minimal information was collected from the CHs, the burden placed on care staff to procure the information was limited, which meant a greater number of CHs were able to supply their menus. The original study design called for full ingredients lists to be provided. This was still requested but was optional, with only eight CHs in the data set providing the more detailed data. An additional strength is the resulting numerical value produced as a result of the scoring system. By producing a number between 1–80, between‐CH comparison was possible. Other novel scoring systems, such as the ProVeg School Plates system, provide a categorical value (Bronze, Silver and Gold) when ranking school menus [36]. Without a numerical scoring system, menus are placed into clusters and therefore are limited in their comparison.

The research has several weaknesses. First, the scoring system has not been externally validated, and although a similar system has been employed in a different paper, the lack of external validation means this tool may lack inter‐rater reliability, thus limiting its reproducibility. The system was initially developed for hospitals and has been adapted for CHs in this study. This limits the scoring system's generalisability in other settings. Additional research is needed to refine and validate this tool for use across various settings. The resulting score produced via the novel scoring system is also arbitrary, suggesting the score cannot categorically represent whether a menu is plant‐forward or not. A further limitation is the length of the menu collection period. The menus were collected across a 4‐month period between August and November 2025 and therefore may not be representative of what a CH provides all year round. Additionally, as this period spans both summer and winter menus in the UK, the menus may not be comparable. Further to this, due to heterogeneity amongst CHs regarding length of menu rotation, only Week 1 of each CH menu was analysed. Individual scores for CHs could vary on a weekly basis, but in the interest of making results comparable, mean scores were not generated across the whole menu rotation. Further research could explore the varying provision of meat, dairy and PB meals on a weekly basis. Another key limitation is the disproportional representation of each region in the United Kingdom. The CHs included in the sample are overwhelmingly based in the South of England. This means that regional differences in the provision of PB and vegetarian meals could not be fully elucidated, and instead any regional differences noted in this study are only tentative. Furthermore, the combined results of the study are also not representative of the United Kingdom as a whole. Future research is needed to capture the degree to which CH menus are plant‐forward in regions that were under‐represented in this study. A final limitation is that the scoring system fails to take into account the proportions of each food item in a given meal, meaning comparisons to NHS guidelines regarding the consumption of red and processed meat cannot be accurately made.

## 5 Conclusion

As determined through the use of a scoring system to calculate the degree to which a menu is plant‐forward, UK CHs, much like hospitals [16], currently do not provide plant‐forward menus. Specifically, the provision of fully PB meals is limited within the studied data set. Although provision of vegetarian meals is common in CHs, entirely PB meals appear to be offered infrequently. Red and processed meat are regularly offered, and more research is required to understand whether the current offerings exceed NHS guidelines on their consumption. Despite this gap, it can still be recommended that CHs should reduce the provision of red and processed meats in favour of PBPs, white meat and fish, which have lower environmental footprints and potentially improved health outcomes. Future research should explore decision‐making about meal choices in CHs.

## Author Contributions

**Saffron Whyton:** conceptualisation, methodology, data collection, data analysis, writing – original draft. **Lisa Methven:** conceptualisation, methodology, writing – reviewing and editing, co‐supervision. **Kathrin Cohen Kadosh:** conceptualisation, methodology, writing – reviewing and editing, co‐supervision. **Victoria Tischler:** conceptualisation, methodology, writing – reviewing and editing, co‐supervision.

## Ethics Statement

This study did not require ethical review due to the absence of human participants. The project was registered with the University of Surrey Ethics Committee (Review Reference: 2025‐2592/2494‐1).

## Conflicts of Interest

The authors declare no conflicts of interest.

## Data Availability

The data supporting this study are available upon request from the corresponding author.
